# Secondary hyperparathyroidism: what we know and what we are still learning

**DOI:** 10.1590/2175-8239-JBN-2025-E008en

**Published:** 2025-06-13

**Authors:** Karina Litchteneker, Sérgio Gardano Elias Bucharles, Fellype de Carvalho Barreto

**Affiliations:** 1 Universidade Federal do Paraná, Departamento de Clínica Médica, Disciplina de Nefrologia, Toledo, PR, Brazil.; 2 Universidade Federal do Paraná, Departamento de Clínica Médica, Disciplina de Nefrologia, Curitiba, PR, Brazil.; 3 Universidade Federal do Paraná, Complexo Hospital de Clínicas, Serviço de Nefrologia, Curitiba, PR, Brazil.

The Chronic Care Model (CCM), a method currently used in Brazil to address chronic kidney disease (CKD) within the Unified Health System, is based on the social determinants of health proposed by Dahlgren and Whitehead in 1991. The CCM proposes different actions for distinct population levels, ranging from health promotion for the general population to individualized management of complex and multimorbid patients. In this context, clinical epidemiology is essential for the management of CKD, as it may identify etiological factors (enabling promotion and prevention actions) as well as aggravating factors and prognostics, allowing for individualized approaches^1^.

One of the main disorders within the scope of CKD is mineral and bone disorder (CKD-MBD). Among the manifestations related to this true syndrome, secondary hyperparathyroidism (SHPT) stands out as one of the most prevalent, and at the same time, as a contributor to complications. Throughout the stages of CKD, several metabolic abnormalities contribute to the progressive elevation of serum parathyroid hormone (PTH) levels, such as hyperphosphatemia, a tendency toward hypocalcemia, decreased calcitriol synthesis, and skeletal resistance to PTH, among others^2^. In general, from stage G3 CKD onwards, serum PTH levels progressively increase, which over time leads to bone alterations as part of the renal osteodystrophy spectrum, known as osteitis fibrosa, and cardiovascular changes, such as ectopic calcification and cardiomyopathy. These changes contribute to the loss of bone mass and quality, and consequently to an increased risk of fragility fractures and cardiovascular mortality^2^.

Data from Brazilian nephrology surveys on renal replacement therapy (RRT) show alarming percentages of patients (on average, 18%) with PTH levels outside therapeutic targets^3,4,5^. These results suggest that, despite greater access, pharmacological treatment of SHPT still remains suboptimal^3–5^.

In addition, data from the DOPPS study show that although mean PTH levels decrease in the first few months after the initiation of RRT, they return to considerably higher levels in the following months – especially among individuals of African descent – even with the prescription of calcimimetics and injectable analogues of active forms of vitamin D^6^. Thus, SHPT may be considered a serious global health issue, with high rates even in countries with better socioeconomic conditions. Furthermore, racial factors, such as African ancestry, seem to negatively impact the metabolic control of CKD-MBD^4–6.^

A study recently published in the Brazilian Journal of Nephrology (BJN) by the Department of Mineral and Bone Metabolism Disorders of the Brazilian Society of Nephrology confirms that at least 9% of patients on RRT – that is, approximately 23,000 patients – have PTH levels > 1,000 pg/mL, the cutoff point used to identify severe forms of SHPT^7^. It is noteworthy that only 2.7% of these individuals underwent parathyroidectomy. Among patients with indications for this surgical procedure, the waiting time exceeded two years in 28% of cases. This highlights that access to surgical treatment for the more severe forms of the disease is still quite limited in our country, reinforcing the importance of early and effective treatment of SHPT, as well as the identification of risk factors related to its worsening in the early stages of RRT^7^.

Although Brazilian data remain scarce, this edition of the BJN presents the study entitled “Serum Parathyroid Hormone Trajectory During the First Year of Hemodialysis: A Roadmap to Severe Hyperparathyroidism,” by Duque E. et al., involving 1,973 participants. The study identified that high PTH levels at the onset of hemodialysis are associated with equally high levels of this hormone after one year, indicating that inadequate management of MBD during the conservative treatment phase of CKD exposes patients to the risk of future parathyroidectomy, even after the initiation of RRT^8^. As observed in previous studies^6^, individuals with PTH levels > 600 pg/mL after 12 months on dialysis are generally younger, non-white, with a low prevalence of diabetes mellitus, and belonging to low socioeconomic groups. Elevated serum levels of alkaline phosphatase and PTH itself (i.e. > 600 pg/ml) at the onset of hemodialysis were independent predictors of difficulty in controlling^6^.

Data on the treatment of CKD-MBD during the intermediate stages of CKD are still quite scarce and could represent an interesting starting point for investigating why a significant proportion of patients start RRT with high PTH levels. Are more regular assessments of PTH levels and other CKD-MBD biomarkers needed during conservative treatment? Is there a shortage of specific medications for treatment? How are these drugs accessed and prescribed? Would early diagnosis and treatment of CKD-MBD reduce morbidity and mortality in chronic kidney disease patients on dialysis? It is known that high levels of PTH in incident dialysis patients are associated with greater difficulty in controlling SHPT in the long term. Additionally, elevated PTH levels and the significant number of dialysis patients awaiting parathyroidectomy indicate that SHPT control remains a major challenge. Therefore, it is essential to maintain a continuous focus on the quality of RRT and adequate monitoring of SHPT, directing efforts not only toward prescription but also toward therapeutic adherence, including the involvement of a multidisciplinary team to guide lifestyle changes, such as adequate calcium and phosphorus intake.

In terms of prospects, data on the epidemiological relevance of MBD in Brazil, presented in the study by Duque E. et al., add to the updates of national and international Nephrology guidelines^4,5^, with the aim of promoting the development of a coherent medical approach to reduce clinical outcomes, such as fractures, cardiac events, and mortality, ultimately improving each patient’s prognosis.
