# Supplementary material for: Secondary hyperparathyroidism: what we know and what we are still learning
Source: J Bras Nefrol. 2025 Jun 13;47(2):e2025E008. doi: 10.1590/2175-8239-JBN-2025-E008en (PMC12172541; doi:10.1590/2175-8239-JBN-2025-E008en)
Supplement: Translation

# Translation: Hiperparatireoidismo secundário: o que sabemos e o que ainda estamos aprendendo

**DOI:** 10.1590/2175-8239-JBN-2025-E008pt

**Authors:** Karina Litchteneker, Sérgio Gardano Elias Bucharles, Fellype de Carvalho Barreto

**Affiliations:** 1 Universidade Federal do Paraná, Departamento de Clínica Médica, Disciplina de Nefrologia, Toledo, PR, Brasil.; 2 Universidade Federal do Paraná, Departamento de Clínica Médica, Disciplina de Nefrologia, Curitiba, PR, Brasil.; 3 Universidade Federal do Paraná, Complexo Hospital de Clínicas, Serviço de Nefrologia, Curitiba, PR, Brasil.

O Modelo de Atenção às Condições Crônicas (MACC), método vigente no Brasil para o enfrentamento da doença renal crônica (DRC) no âmbito do Sistema Único de Saúde, fundamenta-se nos determinantes sociais da saúde propostos por Dahlgren e Whitehead em 1991. O MACC propõe diferentes ações para distintos níveis populacionais, desde a promoção da saúde para a população geral até o manejo individualizado do paciente complexo e multicomórbido. Nesse contexto, a epidemiologia clínica é fundamental para o manejo da DRC, uma vez que pode apontar fatores etiológicos (viabilizando ações de promoção e prevenção), bem como agravantes e prognósticos, permitindo condutas individuais^1^.

Um dos principais distúrbios no âmbito da DRC é o distúrbio mineral-ósseo (DMO-DRC). Entre as manifestações relacionadas a essa verdadeira síndrome, o hiperparatireoidismo secundário (HPTs) destaca-se como uma das mais prevalentes e, ao mesmo tempo, como promotora de complicações. Ao longo dos estágios da DRC, diversas alterações metabólicas concorrem para a progressiva elevação dos níveis séricos de paratormônio (PTH), como a hiperfosfatemia, a tendência à hipocalcemia, a menor síntese de calcitriol e a resistência óssea ao PTH, entre outras^2^. De maneira geral, a partir do estágio G3 da DRC, os níveis séricos de PTH elevam-se progressivamente, o que, ao longo do tempo, acarreta alterações ósseas como parte do espectro da osteodistrofia renal, denominada osteíte fibrosa, e alterações cardiovasculares, como calcificação ectópica e miocardiopatia. Tais alterações contribuem para a perda de massa e qualidade ósseas e, consequentemente, para o aumento do risco de fraturas de fragilidade e de mortalidade cardiovascular^2^.

Dados dos censos brasileiros de nefrologia em terapia renal substitutiva (TRS) evidenciam porcentagens alarmantes de pacientes (em média, 18%) com níveis de PTH fora dos alvos terapêuticos^3–5^. Esses resultados sugerem que, a despeito do maior acesso, o tratamento farmacológico do HPTs ainda permanece aquém do ideal^3–5^.

Adicionalmente, dados do estudo DOPPS demonstram que, embora os níveis médios de PTH se reduzam nos primeiros meses após o início da TRS, eles voltam a se elevar consideravelmente nos meses subsequentes, especialmente entre indivíduos afrodescendentes, mesmo com a prescrição de calcimiméticos e análogos injetáveis das formas ativas da vitamina D^6^. Dessa forma, pode-se considerar que o HPTs constitui um grave problema de alcance global, com elevadas taxas mesmo em países com melhores condições socioeconômicas. Ademais, fatores raciais, como a afrodescendência, parecem impactar negativamente o controle metabólico do DMO-DRC^4–6^.

Um estudo recentemente publicado no Jornal Brasileiro de Nefrologia (JBN), pelo Departamento de Metabolismo Mineral e Ósseo da Sociedade Brasileira de Nefrologia, confirma que ao menos 9% dos pacientes em TRS – ou seja, um contingente de aproximadamente 23.000 pacientes – apresentam valores de PTH > 1.000 pg/mL, ponto de corte utilizado para identificar formas graves de HPTs^7^. Ressalta-se que apenas 2,7% desses indivíduos foram submetidos à paratiroidectomia. Dentre os pacientes com indicação para esse procedimento cirúrgico, o tempo de espera foi superior a dois anos em 28% dos casos. Isso evidencia que o acesso ao tratamento cirúrgico nas formas mais graves da doença é ainda bastante limitado em nosso país, reforçando a importância do tratamento precoce e satisfatório do HPTs, bem como da identificação de fatores de risco relacionados ao seu agravamento nas fases iniciais da TRS^7^.

Embora os dados brasileiros permaneçam escassos, esta edição do JBN apresenta o estudo intitulado “*Serum Parathyroid Hormone Trajectory During the First Year of Hemodialysis: A Roadmap to Severe Hyperparathyroidism*”, de Duque E. e cols., com 1973 participantes. O estudo identificou que altos níveis de PTH no início da hemodiálise associam-se a níveis igualmente elevados desse hormônio após 1 ano, indicando que o manejo inadequado do DMO durante a fase de tratamento conservador da DRC expõe os pacientes ao risco de futura paratireoidectomia, mesmo após a instituição da TRS^8^. Como observado em estudos prévios^6^, os indivíduos que apresentam níveis de PTH > 600 pg/mL após 12 meses em diálise são, em geral, mais jovens, não brancos, com baixa prevalência de diabetes mellitus e pertencentes a grupos de baixo padrão socioeconômico. Níveis séricos elevados de fosfatase alcalina e do próprio PTH (*i.e*. > 600 pg/ml) no início da hemodiálise foram preditores independentes de dificuldade no controle^6^.

Dados sobre o tratamento do DMO-DRC nos estágios intermediários da DRC ainda são bastante escassos e poderiam representar um ponto de partida interessante para investigar por que um contingente significativo de pacientes inicia a TRS com níveis elevados de PTH. Faltam avaliações mais regulares dos níveis de PTH e dos demais biomarcadores do DMO-DRC durante o tratamento conservador? Há carência de medicações específicas para o tratamento? Como se dá o acesso e a prescrição desses fármacos? O diagnóstico e o tratamento precoces do DMO-DRC reduziriam a morbidade e a mortalidade dos pacientes renais crônicos na fase dialítica? Sabe-se que altos níveis de PTH em pacientes incidentes em diálise estão associados a maior dificuldade no controle do HPTs a longo prazo. Adicionalmente, os níveis elevados de PTH e o número expressivo de pacientes em diálise aguardando paratireoidectomia indicam que o controle do HPTs continua a representar um grande desafio. Portanto, é fundamental manter o foco contínuo na qualidade da TRS e na vigilância adequada do HPTs, direcionando esforços não apenas à prescrição, mas também à aderência terapêutica, inclusive com a participação da equipe multidisciplinar orientando mudanças de hábitos de vida, como a ingestão adequada de cálcio e fósforo.

Como perspectivas, os dados de relevância epidemiológica do DMO no Brasil, apresentados no estudo de Duque E. e cols. somam-se às atualizações das diretrizes nacionais e internacionais em Nefrologia^4,5^, com o objetivo de fomentar a estruturação de uma conduta médica coerente para a redução de desfechos clínicos, como fraturas, eventos cardíacos e mortalidade, a fim de melhorar o prognóstico de cada paciente.
